# Association of serum uric acid and fasting plasma glucose with cognitive function: a cross-sectional study

**DOI:** 10.1186/s12877-023-03998-9

**Published:** 2023-05-05

**Authors:** Zelin Yuan, Huamin Liu, Rui Zhou, Shanyuan Gu, Keyi Wu, Zhiwei Huang, Qi Zhong, Yining Huang, Haowen Chen, Xianbo Wu

**Affiliations:** 1 Department of Epidemiology, School of Public Health, Guangdong Provincial Key Laboratory of Tropical Diseases, Southern Medical University, Guangzhou, China; 2 Department of Psychiatry, Baiyun Psychiatric Rehabilitation Hospital, No.2 Helong Five Road, Guangzhou, 510445 Guangdong China

**Keywords:** Cognitive function, Fasting plasma glucose, Serum uric acid, Gender differences

## Abstract

**Background:**

The combined effect of serum uric acid (SUA) and blood glucose on cognition has not been explored. This study aimed to examine the separate and combined association of SUA and fasting plasma glucose (FPG) or diabetes mellitus (DM) with cognition in a sample of Chinese middle-aged and elderly population.

**Methods:**

A total of 6,509 participants aged 45 years or older who participated in the China Health and Retirement Longitudinal Study (CHARLS, 2011) were included. The three cognitive domains assessed were episodic memory, mental status, and global cognition (the sum of the first two terms). Higher scores indicated better cognition. SUA and FPG were measured. The participants were grouped based on SUA and FPG quartiles to evaluate their combined associations of cognition with SUA Q1–Q3 only (Low SUA), with FPG Q4 only (High FPG), without low SUA and high FPG levels (Non), and with low SUA and high FPG levels (Both), multivariate linear regression models were used to analyze their association.

**Results:**

Lower SUA quartiles were associated with poorer performance in global cognition and episodic memory compared with the highest quartile. Although no association was found between FPG or DM and cognition, high FPG or DM combined with low SUA levels in women (β_FPG_ = -0.983, 95% CI: -1.563–-0.402; β_DM_ = -0.800, 95% CI: -1.369–-0.232) had poorer cognition than those with low SUA level only (β_FPG_ = -0.469, 95% CI: -0.926–-0.013; β_DM_ = -0.667, 95% CI: -1.060–-0.275).

**Conclusion:**

Maintaining an appropriate level of SUA may be important to prevent cognitive impairment in women with high FPG.

**Supplementary Information:**

The online version contains supplementary material available at 10.1186/s12877-023-03998-9.

## Introduction

With the aging population worldwide, dementia causes a heavy burden on human society [1]. Patients with advanced stages of dementia can be severely disabled, and the prevalence of dementia is steadily increasing. However, specific drugs to treat dementia are still lacking. People with cognitive impairment likely develop dementia decades later [2]. The global prevalence of cognitive impairment increases every year and ranges from 6 to 12%. Preventing cognitive dysfunction could significantly reduce the high prevalence of dementia worldwide [3]. The prevalence of mild cognitive impairment among the Chinese older population over 60 years old reached 15.5% (15.2–15.9) in 2020, with a total population of approximately 38.77 million [4].

Recent studies have linked low serum uric acid (SUA) levels with poor cognitive performance [5]. In a case–control study with random sampling, the mini-mental state examination (MMSE) score was linearly and inversely associated with SUA level [6]. Prospective cohort studies also found that a low baseline SUA level could be a risk factor for multiple domains of cognitive function in older adults [7]. SUA acts as a major antioxidant and reduces the risk of neurodegenerative diseases by protecting neurons from oxidative damages [5, 8].

Considerable evidence has shown that blood glucose affects cognitive function [9, 10]. Diabetes mellitus (DM), glycemic control, and DM duration are related to cognitive dysfunction [11]. A previous study reported that fasting plasma glucose (FPG) levels are associated with executive function, but not other cognitive domains. Therefore, high glucose levels may concur to the multifactorial pathogenesis of cognitive dysfunction. Appropriate blood glucose regulation may thus improve the protection of executive function in cognitively impaired older adults [12].

Mice lacking SUA transporters in the gut likely develop metabolic syndrome and hyperuricemia, indicating evidence of the causal connection between high levels of SUA and blood glucose [13]. Another study found that hyperinsulinemia inhibits renal SUA metabolism and leads to elevated SUA levels, suggesting an association between SUA and hypoglycemia [14]. Other studies reported no significant relationship between SUA and FPG [15]. A previous work on the China Health and Retirement Longitudinal Study (CHARLS) found that men and women showed an L-shaped relationship between SUA and blood glucose [16]. Furthermore, the association of SUA and FPG with cognitive function among older adults remains unclear.

This study aimed to explore the association of FPG, SUA, and their combination with cognitive function and to predict the progression of cognitive function impairment to a certain extent.

## Materials and methods

### Study population

CHARLS is a full-scale, nationally representative longitudinal survey of middle-aged and older adults (≥ 45 years old) conducted by the National Institute of Development at Peking University. The survey used data from the 2011 national baseline survey with 17,705 participants. The exact content of CHARLS was detailed elsewhere [17]. Participants who were < 45 years old (*n* = 363), had a proxy interview (*n* = 1212), did not complete a cognitive assessment (*n* = 6468), had a history of brain damage or intellectual disability at baseline (*n* = 130), and had missing FPG or SUA data (*n* = 3023) were excluded from our study. Finally, a total of 6509 participants were included for analysis (Table 1).

**Table 1:** Baseline characteristics

	All participants (*n* = 6509)	Non (*n* = 1117)	Low SUA (*n* = 3764)	High FPG (*n* = 511)	Both (*n* = 1117)	*P* value
Age, years [Q1–Q3]	57 [51–64]	58 [51–65]	56 [49–63]	59 [52–66]	58 [53–64]	** < 0.001**
Male (n, %)	3257 (50.03)	565 (50.58)	1830 (48.62)	250 (48.92)	612 (54.79)	**0.004**
FPG, mmol/L	5.69 [5.25–6.30]	5.55 [5.17–5.86]	5.44 [5.10–5.77]	6.93 [6.54–8.09]	7.16 [6.59–8.65]	** < 0.001**
UA, mg/dL	4.36 [3.62–5.22]	5.93 [5.13–6.62]	3.97 [3.42–4.55]	6.05 [5.25–6.80]	4.05 [3.48–4.64]	** < 0.001**
Educational level						0.248
Primary school or lower	3895 (14.86)	662 (59.27)	2261 (60.07)	293 (57.34)	679 (60.79)	
Middle school	1647 (25.30)	275 (24.62)	949 (25.21)	129 (25.24)	294 (26.32)	
High school or above	967 (59.84)	180 (16.11)	554 (14.72)	89(17.42)	144 (12.89)	
Currently married	5917 (90.90)	1003 (89.79)	3448 (91.60)	463 (90.61)	1003 (89.79)	0.132
Smoking status						** < 0.001**
Current	2043 (31.39)	342 (30.62)	1192 (31.67)	135 (26.42)	374 (33.48)	
Former	614 (9.43)	115 (10.30)	301 (8.00)	64 (12.5)	134 (12.00)	
Never	3852 (59.18)	660 (59.09)	2270 (60.31)	312 (61.06)	609 (54.52)	
Alcohol consumption						0.057
Current	2274 (34.94)	401 (35.90)	1294 (34.38)	171 (33.46)	408 (36.53)	
Former	330 (5.07)	68 (6.09)	170 (4.52)	36 (7.05)	56 (5.01)	
Never	3905 (59.99)	648 (58.01)	2300 (61.11)	304 (59.49)	653 (58.46)	
Global Cognition	16.73 ± 4.25	17.06 ± 4.33	16.69 ± 4.22	16.90 ± 4.30	16.50 ± 4.22	**0.012**
Episodic Memory	8.17 ± 2.97	8.41 ± 3.07	8.14 ± 2.94	8.23 ± 3.12	8.02 ± 2.89	**0.013**
Mental Status	8.56 ± 2.31	8.65 ± 2.28	8.55 ± 2.31	8.67 ± 2.29	8.49 ± 2.34	0.775
BMI, kg/m^2^	23.48 [21.23–26.14]	24.22 [21.91–26.95]	22.96 [20.86–25.41]	25.33 [22.70–27.86]	23.99 [21.68–26.65]	** < 0.001**
DM	1081 (16.61)	47 (4.21)	103 (2.74)	273 (53.42)	658 (58.91)	** < 0.001**
Hypertension	1667 (25.61)	363 (32.50)	746 (19.82)	232 (45.40)	326 (29.19)	** < 0.001**
Dyslipidemia	698 (10.72)	134 (12.00)	308 (8.18)	108 (21.14)	148 (13.25)	** < 0.001**
Kidney disease	435 (6.68)	80 (7.16)	268 (7.12)	27 (5.28)	60 (5.37)	0.098
Liver disease	260 (3.99)	49 (4.39)	142 (3.77)	24 (4.70)	45 (4.03)	0.662
Stroke	117 (1.80)	26 (2.33)	52 (1.38)	20 (3.91)	19 (1.70)	** < 0.001**
Gastrointestinal disease	1436 (22.06)	212 (18.98)	895 (23.78)	79 (15.46)	250 (22.38)	** < 0.001**
Heart diseases	809 (12.43)	119 (10.65)	423 (11.24)	96 (18.79)	171 (15.31)	** < 0.001**
Treatment for kidney disease	244 (3.75)	47 (4.21)	151 (4.01)	12 (2.35)	34 (3.04)	0.128
Diabetes treatment	281 (4.32)	19 (1.70)	44 (1.17)	63 (12.33)	155 (13.88)	** < 0.001**

*Abbreviations: FPG* Fasting plasma glucose, *SUA* Serum uric acid, *BMI* Body mass index, *DM* Diabetes mellitus. *P* values < 0.05 were highlighted in bold

CHARLS was approved by the Peking University Ethics Review Committee. Informed consent was sought from all participants.

### Cognitive assessment

The participants were given cognitive measures (including the two cognitive areas of episodic memory and mental status) by uniformly trained investigators. The investigators read a list of Chinese words to the participants. After which, the participants immediately enumerated the nouns that they heard and then recalled as many nouns as possible after 5 min (delayed recall). Episodic memory was generally defined as the sum of immediate and delayed recall scores, ranging from 0 to 20. Mental status was assessed using the Telephone Interview of Cognitive Status (TICS) questionnaire, which is an adequate and reasonable method to capture the mental status or integrity of a person. The participants answered the following questions: subtract 7 several times in a row from 100 (up to five times); name the date of the day (day, week, month, year, and season), and redraw the picture he/she had been shown. The right answers were summed up to a single TICS score ranging from 0 to 11. The global cognition score was determined by summing up the episodic memory and TICS scores and ranged from 0 to 31.

### Measurements of SUA and FPG

Blood samples were collected from the participants after they fasted overnight by uniformly trained staff of the Chinese Center for Disease Control and Prevention following standard protocols. FPG level was determined at the central study laboratory in Beijing. SUA level (mg/dL) was analyzed using SUA Plus method, and FPG was measured using an enzymatic colorimetric test. The participants were divided into groups based on their SUA and FPG quartiles. They were also divided into the following groups to evaluate the effect of interplay between SUA and FPG on cognitive function with SUA Q1–Q3 only (Low SUA), with FPG Q4 only (High FPG), without low SUA and high FPG levels (Non), and with low SUA and high FPG levels (Both).

### Covariates

Several potential covariates were collected including age, sex, body mass index (BMI), marital status (currently married or not), educational level (primary school or lower, middle school, and high school or above), smoking status (current, former, and never), alcohol consumption (current, former, and never), diagnosis of hypertension, diabetes, dyslipidemia, kidney disease, stroke, heart diseases, liver disease, gastrointestinal disease, psychiatric problems, and treatment of kidney disease and diabetes (including taking Chinese or western traditional medicine and taking insulin injections or other treatments).

### Statistical analysis

Continuous variables that were not normally distributed or had heterogeneity of variance were represented by median [interquartile range]. The remaining continuous variables were represented by means (standard deviations). Categorical variables were represented by percentages. Depending on the situation, Kruskal–Wallis test or analysis of variance was used for continuous variables. Chi-square test was used for classified variables. Multivariable linear regression models were used to examine the association between SUA and FPG levels and cognitive scores.

Subgroup analysis of sex was performed because sex was associated with lower scores on cognitive testing in the multivariate model. The statistical significance of the interactions was assessed by adding a multiplicative term to the linear regression model. Sensitivity analysis was conducted to determine the robustness of the primary results. All data were analyzed using STATA version 14 (StataCorp LP, College Station, Texas, USA).

## Results

### Baseline characteristics

The baseline characteristics of the study population are listed in Table 1. The Non, Low SUA, High FPG, and Both groups had 1117, 3764, 511, and 1117 participants, respectively. The average age of the total population was 57 [51–64] years, and the male population accounted for 50.03%. Statistically significant differences were found in age, sex, FPG, UA, smoking status, BMI, DM, hypertension, dyslipidemia, stroke, heart diseases, gastrointestinal disease and diabetes treatment among all groups.

### Association between SUA/FPG level and cognitive function

First, the association of SUA level with different cognitive domains was examined. After adjusting for age, sex, BMI, smoking status, hypertension, dyslipidemia, stroke, heart diseases, kidney disease, liver disease, gastrointestinal disease, diabetes and diabetes treatment, the participants in the lower SUA quartiles had incrementally lower cognitive scores (global cognition and episodic memory) than those in the highest quartile groups. The β values (95% CI) of the participants were -0.460 (-0.768, -0.153) for the SUA Q2 group and -0.420 (-0.731, -0.109) for SUA Q1 group in global cognition scores and -0.339 (-0.557, -0.122) for the SUA Q2 group and -0.311 (-0.531, -0.091) for the SUA Q1 group in episodic memory compared with those in the SUA Q4 group (Table 2).

**Table 2:** Association between quartiles of SUA and cognitive function

Variable	Global Cognition β (95% CI)	Episodic Memory β (95% CI)	Mental Status β (95% CI)
SUA Q4	Ref	Ref	Ref
SUA Q3	-0.256 (-0.561, 0.050)	-0.194 (-0.410, 0.022)	-0.062 (-0.229, 0.105)
SUA Q2	**-0.460 (-0.768, -0.153)**	**-0.339 (-0.557, -0.122)**	-0.121 (-0.290, 0.047)
SUA Q1	**-0.420 (-0.731, -0.109)**	**-0.311 (-0.531, -0.091)**	-0.109 (-0.279, 0.061)
*P* for trend	**0.004**	**0.003**	0.159
Age	**-0.117 (-0.130, -0.104)**	**-0.079 (-0.088, -0.070)**	**-0.038 (-0.045, -0.031)**
Sex	**-1.304 (-1.586, -1.023)**	**-0.223 (-0.422, -0.024)**	**-1.082 (-1.236, -0.927)**
BMI	**0.094 (0.064, 0.124)**	**0.050 (0.029, 0.072)**	**0.044 (0.027, 0.060)**
Smoke	-0.130 (-0.285, 0.024)	-0.031 (-0.140, 0.078)	**-0.099 (-0.184, -0.015)**
DM	0.042 (-0.285, 0.368)	-0.001 (-0.231, 0.231)	0.042 (-0.137, 0.221)
Hypertension	**0.328 (0.054, 0.602)**	**0.263 (0.070, 0.457)**	0.065 (-0.085, 0.215)
Dyslipidemia	-0.314(-0.691, 0.063)	-0.273 (-0.540, -0.006)	**-**0.041 (-0.248, 0.165)
Stroke	0.095 (-0.734, 0.923)	0.148 (-0.438, 0.734)	-0.053 (-0.506, 0.400)
Heart diseases	-0.249 (-0.600, 0.103)	-0.092 (-0.341, 0.156)	-0.156 (-0.348, 0.036)
Kidney disease	0.206 (-0.230, 0.642)	0.040 (-0.268, 0.348)	0.166 (-0.073, 0.404)
Liver disease	-0.405 (-0.955, 0.144)	-0.327 (-0.716, 0.061)	-0.078 (-0.378, 0.222)
Gastrointestinal disease	**0.327 (0.063, 0.590)**	0.176 (-0.010, 0.363)	**0.150 (0.006, 0.295)**
Diabetes treatment	-0.046 (-0.656, 0.564)	-0.113 (-0.544, 0.319)	0.066 (-0.267, 0.400)

Adjusted for age, sex, BMI, smoking status, hypertension, dyslipidemia, stroke, heart diseases, kidney disease, liver disease, gastrointestinal disease, diabetes and diabetes treatment

*Abbreviations: SUA* Serum uric acid, *BMI* Body mass index, *DM* Diabetes mellitus. β_s_ 95% CI without 0, or *P* values < 0.05 were highlighted in bold

The association of FPG level with different cognitive domains was also examined. After adjusting for age, sex, BMI, smoking status, hypertension, dyslipidemia, stroke, heart diseases, and diabetes treatment, no statistical association was found between FPG and cognitive score (Table 3).

**Table 3:** Association between quartiles of FPG and cognitive function

Variable	Global Cognition β (95% CI)	Episodic Memory β (95% CI)	Mental Status β (95% CI)
FPG Q1	Ref	Ref	Ref
FPG Q2	0.165 (-0.138, 0.469)	0.092 (-0.123, 0.306)	0.073 (-0.092, 0.239)
FPG Q3	-0.079 (-0.384, 0.227)	-0.048 (-0.264, 0.168)	-0.031 (-0.198, 0.136)
FPG Q4	-0.096 (-0.409, 0.216)	-0.052 (-0.273, 0.169)	-0.044 (-0.215, 0.127)
*P* for trend	0.296	0.411	0.395
Age	**-0.114 (-0.127, -0.101)**	**-0.077 (-0.086, -0.068)**	**-0.037 (-0.044, -0.030)**
Sex	**-1.331 (-1.612, -1.050)**	**-0.241 (-0.440, -0.042)**	**-1.090 (-1.243, -0.936)**
BMI	**0.107 (0.077, 0.137)**	**0.058 (0.036, 0.079)**	**0.049 (0.033, 0.065)**
Smoke	-0.132 (-0.286, 0.022)	-0.034 (-0.143, 0.075)	**-0.098 (-0.182, -0.014)**
Hypertension	0.271 (-0.002, 0.543)	**0.226 (0.033, 0.418)**	0.045 (-0.104, 0.193)
Dyslipidemia	-0.307 (-0.683, 0.068)	**-0.277 (-0.543, -0.011)**	**-**0.031 (-0.236, 0.175)
Stroke	0.031 (-0.789, 0.852)	0.101 (-0.480, 0.681)	-0.069 (-0.518, 0.379)
Heart diseases	-0.183 (-0.530, 0.164)	-0.060 (-0.306, 0.186)	-0.123 (-0.312, 0.067)
Diabetes treatment	0.048 (-0.514, 0.609)	-0.078 (-0.476, 0.319)	0.126 (-0.181, 0.433)

Adjusted for age, sex, BMI, smoking status, hypertension, dyslipidemia, stroke, heart diseases, diabetes treatment

*Abbreviations: FPG* Fasting plasma glucose, *BMI* Body mass index. β_s_ 95% CI without 0, or *P* values < 0.05 were highlighted in bold

### Combined association of FPG and SUA with cognitive function

The combined association of SUA and FPG with cognitive function was significant in global cognition and episodic memory after adjusting for additional confounders (Table 4). After stratification by sex, the combination of lower SUA and higher FPG in females was associated with poorer performance in global cognition (β = -0.983, 95% CI: -1.563–-0.402) and episodic memory (β = -0.666, 95% CI: -1.072–-0.259). The two models with different adjustments were significant (Table 5).

**Table 4:** Association between combination of FPG and SUA quartiles and cognitive function

All participants	Global Cognition		Episodic Memory		Mental Status	
	Model 1 β (95% CI)	Model 2 β (95% CI)	Model 1 β (95% CI)	Model 2 β (95% CI)	Model 1 β (95% CI)	Model 2 β (95% CI)
	Model 1	Model 2	Model 1	Model 2	Model 1	Model 2
Non	Ref	Ref	Ref	Ref	Ref	Ref
Low SUA	**-0.346 (-0.642, -0.050)**	**-0.383 (-0.683, -0.083)**	**-0.286 (-0.496, -0.077)**	**-0.305 (-0.517, -0.093)**	-0.060 (-0.222, 0.102)	-0.078 (-0.242, 0.086)
High FPG	-0.111 (-0.568, 0.346)	-0.154 (-0.618, 0.309)	-0.145 (-0.469, 0.178)	-0.147 (-0.475, 0.181)	0.034 (-0.216, 0.284)	-0.007 (-0.261, 0.246)
Both	**-0.551 (-0.912, -0.190)**	**-0.545 (-0.917, -0.173)**	**-0.390 (-0.645, -0.135)**	**-0.383 (-0.646, -0.120)**	-0.161 (-0.358, 0.037)	-0.163 (-0.366, 0.041)

Model 1: adjusted for age, sex, BMI

Model 2: adjusted for age, sex, BMI, smoking status, hypertension, dyslipidemia, stroke, heart diseases, kidney disease, liver disease, gastrointestinal disease and diabetes treatment

*Abbreviations: FPG* Fasting plasma glucose, *SUA* Serum uric acid, *BMI* Body mass index. β_s_ 95% CI without 0 were highlighted in bold

**Table 5:** Association between combination of FPG and SUA quartiles and cognitive function (stratified by sex)

	Global Cognition β (95% CI)		Episodic Memory β (95% CI)		Mental Status β (95% CI)	
**Male**	Model 1	Model 2	Model 1	Model 2	Model 1	Model 2
Non	Ref	Ref	Ref	Ref	Ref	Ref
Low SUA	-0.323 (-0.711, 0.065)	-0.314 (-0.705, 0.078)	**-0.324 (-0.601, -0.046)**	**-0.317 (-0.598, -0.037)**	-0.001 (-0.216, 0.216)	0.004 (-0.215, 0.222)
High FPG	-0.098 (-0.704, 0.509)	-0.120 (-0.733, 0.494)	-0.204 (-0.638, 0230)	-0.201 (-0.641, 0.238)	0.106 (-0.231, 0.444)	0.082 (-0.260, 0.423)
Both	-0.227 (-0.691, 0.238)	-0.182 (-0.657, 0.293)	-0.194 (-0.526, 0.138)	-0.158 (-0.498, 0.182)	-0.033 (-0.291, 0.226)	-0.024 (-0.288, 0.241)
**Female**						
Non	Ref	Ref	Ref	Ref	Ref	Ref
Low SUA	**-0.458 (-0.908, -0.008)**	**-0.469 (-0.926, -0.013)**	-0.302 (-0.617, 0.014)	-0.298 (-0.618, 0.022)	-0.156 (-0.398, 0.086)	-0.171 (-0.417, 0.075)
High FPG	-0.046 (-0.733, 0.641)	-0.197 (-0.896, 0.503)	-0.046 (-0.528, 0.436)	-0.115 (-0.604, 0.375)	0.001 (-0.369, 0.370)	-0.082 (-0.459, 0.294)
Both	**-0.900 (-1.459, -0.342)**	**-0.983 (-1.563, -0.402)**	**-0.619 (-1.011, -0.227)**	**-0.666 (-1.072, -0.259)**	-0.281 (-0.582, 0.019)	**-0.317 (-0.629, -0.005)**
*P* for interaction	**0.047**	**0.046**	0.072	0.078	0.193	0.169

Model 1: adjusted for age, BMI, hypertension

Model 2: adjusted for age, BMI, smoking status, hypertension, dyslipidemia, stroke, heart diseases, kidney disease, liver disease, gastrointestinal disease and diabetes treatment

*Abbreviations: FPG* Fasting plasma glucose, *SUA* Serum uric acid, *BMI* Body mass index. β_s_ 95% CI without 0, or *P* values < 0.05 were highlighted in bold

### Sensitivity analysis

According to the American diabetes association, diabetes is diagnosed if any of the following criteria are met: (1) FPG > 7.0 mmol/L; (2) random blood glucose > 11.1 mmol/L; (3) HBA1c > 6.5%; (4) self-reported diabetes diagnosed by physicians; and (5) intake of antidiabetic drugs or insulin therapy [18]. Although the association between DM and cognitive performance was not statistically significant in either men or women (Tables S2 and Table S3), in the female group, the global cognition performance and episodic memory performance of patients with DM and low SUA was poorer than those without DM but low SUA (Table S4), similar to the main results. In addition, the results of the association of SUA and FPG as continuous variables with cognitive scores are presented in the supplementary material (Tables S5 and Table S6).

## Discussion

This study examined the relationship of SUA, FPG levels, and DM to cognitive function among middle-aged and older Chinese population. A low SUA level was associated with poor cognition, but no significant relationship was observed between FPG or DM and cognitive function. Higher FPG level combined with lower SUA level was related to poorer cognitive performance among female participants. Similar results were found in patients with DM and low SUA. The association remained significant after adjusting for a wide array of health-related variables.

The findings of our research are consistent with those of previous studies, which demonstrated the association between low SUA levels and poor cognitive function. For example, a prospective population cohort study of 4,618 participants aged 55 years and above found that higher SUA levels were associated with a lower risk of dementia; as such, a high SUA level was a protective factor for cognitive function [19]. A meta-analysis reported that SUA levels were higher in healthy controls without dementia but lower in patients with dementia [20]. This result is consistent with our findings, that is, a low SUA level was associated with poor cognition. The insignificant association between FPG and cognitive function is also in line with previous results. A Finnish National 2000 Health Examination Survey and a subsequent 11-year follow-up study (baseline included 3695 participants, mean age 49.3, range 30–86 years, 55.5% women, participants who were treated with insulin or unknown diabetes medication were excluded) also found that FPG levels were not associated with cognitive function [21]. However high blood sugar levels can cause cognitive impairment in individuals with diabetes; cognitive decline occurs in areas such as memory, orientation, and executive function [22]. The relationship between plasma glucose, especially outside of diabetes and within the reference range, to cognitive functions remains less clear. A previous study of elderly people (≥ 55 years of age) in a Chinese community found that normal FPG, impaired FPG, and glucose in a diabetic state were not associated with the degree of cognitive dysfunction (as graded by MMSE scores) [23]. Moreover, blood glucose was not associated with cognitive performance in participants without cognitive impairment [12].These inconsistencies in the association between plasma glucose and cognition could be attributed to the interaction between FPG and SUA.

As the metabolic end product of purines [24], the homeostasis of SUA is jointly balanced by endogenous production, exogenous supply, excretion and reabsorption. Xanthine oxidoreductase is the enzyme directly responsible for the conversion of purine bases to SUA. The endogenous production of SUA mainly occurs in tissues with high expression of xanthine oxidoreductase. In humans, the epithelial cells of liver, gastrointestinal tract, kidney and lactation mammary gland have the expression of this enzyme [25], and the organs with the highest expression activity are the liver and small intestine [26]. The exogenous supply of SUA is mainly derived from dietary behaviors such as high-purine foods and alcohol intake, which cause an increase in SUA of about 1-2 mg/dL [27]. SUA excretion and reabsorption efficiencies depend on the associated transport system. SUA is mainly excreted by the kidneys (about 70%), and the rest (about 30%) is excreted by the feces of the intestine. Twice reabsorptions of uric acid by the kidneys, resulting in 5 to 15% of the initial uric acid being excreted in the urine [28]. The difference in the normal physiological range of SUA between the sexes may be due to potential sex differences in these mechanisms. One study found that plasma xanthine oxidoreductase activity was higher in females than in males [29]. And the increase in SUA caused by a high-purine diet is more likely to occur in men [30]. The higher prevalence of gastrointestinal disease in Chinese elderly was found in women in this study (Table S1). In addition, there were gender differences in renal function impairment [31]. These potential sex differences in the mechanisms by which SUA maintains homeostasis provide an important basis for our sex-stratified analysis.

The potential risk effect of low SUA on cognitive domains may be explained by several mechanisms. Many studies have demonstrated the antioxidant properties of SUA over the decades. Urate accounts for about half of the antioxidant capacity of human plasma, and its antioxidant properties are as powerful as ascorbic acid [27, 28]. The main function of SUA is to remove reactive oxygen species and peroxynitrite; urate also protects the human blood from iron-mediated oxidation of ascorbate [32, 33]. In addition, under a phylogenetic perspective, the inability to catabolize uric acid might have conferred an advantage against age-related diseases mediated by high circulating SUA levels [34]. The intricate mechanisms of SUA in oxidative stress are important. The SUA level may affect the oxidative activity in the brain to a certain extent, and oxidative stress is a significant cause of cognitive dysfunction [35]. SUA is a potent antioxidant that has been studied for potential neuroprotective treatment [36]. The mechanism of action of SUA appears pleiotropic and is not only confined to a redox paradigm. Another study found that lower levels of SUA were associated with lower brain metabolism related to cognitive disorder [37]. These findings are the basic hypothesis of the research on SUA and cognitive function.

The combination of low SUA and high FPG or DM was associated with poor cognitive function among the female participants. A low level of SUA is a potential risk factor for the nervous system due to its antioxidant properties. Hyperglycemia increases the body's additional redox stress burden [38]. In animal models of DM, hyperglycemia induces a redox imbalance (an increase in the NADH/NAD^+^ ratio due to the oxidation of NADH to NAD^+^), which in turn adversely affects vascular and neurological function [39]. This redox stress depletes the body of other antioxidants [40]. Therefore, lack of SUA is more dangerous during hyperglycemia, and the co-existence of the two potential cognitive risk factors may result in poor cognitive outcome. This finding suggests that a moderate increase in SUA is associated with better cognitive function in female patients with higher blood glucose levels. The high glucose state increases the number of oxygen free radicals, induces endothelial cell apoptosis, restricts cerebrovascular production, and affects cerebral blood supply [41], leading to neurological dysfunction [42, 43]. In hyperglycemia, endothelial nitric oxide synthase is susceptible to uncoupling. When uncoupling occurs, NAD(P)H reacts with O_2_ and endothelial cells produce superoxide (O_2_•) instead of protective endothelial NO [44]. NO protects the antioxidant properties of SUA [45],so decreased NO production during hyperglycemia may lead to a reduced antioxidant contribution of SUA. This phenomenon is a possible explanation for the lack of protective cognitive effect of high FPG combined with high SUA.

The association of high FPG and low SUA with poor cognition was observed in women only, indicating that elderly women with high FPG are more in need of high SUA for antioxidant. Corroborating this point of view, a study in premenopausal women after hysterectomy, oviduct, and ovariectomy reported that the gene expression of superoxide dismutase and glutathione peroxidase is estrogen dependent [46]. Animal experiments in rats showed increased activity of NADPH oxidase (which promotes reactive oxygen species generation) when estrogen is absent [47]. Decreases in estrogen level can change the expression of important oxidative stress-related enzymes, causing heavy oxidative stress among elderly women. The APOE4 allele is the most important genetic factor that increases the risk of Alzheimer's disease [48]. An animal model study found that female APOE4 carriers had higher levels of oxidative stress in their brains, especially at synaptic terminals [49]. A human RNAseq analysis also showed that oxidative stress-related genes were highly expressed in female APOE4 carriers [50]. Functional magnetic resonance imaging (MRI) showed that female APOE4 carriers had weaker brain connectivity in the precuneus and posterior cingulate cortex compared with male APOE4 carriers [51]. Studies of APOE4 support indicated that the female brain had a higher risk of oxidative stress than the male brain. Female diabetic rats had significantly higher levels of NADPH oxidase 1 and NADPH oxidase 4 than female non-diabetic rats and male diabetic or non-diabetic rats [52]. The results suggest that high glucose level and female gender are important risk factors for oxidative stress. In addition, white matter lesions are a pathological substrate for cognitive impairment [53], as individuals with cognitive impairment were found to have more periventricular white matter hyperintensities (WMH) on the MRI scans [54]. People with higher FPG had more WMH than those with normal FPG [55]. The difference in the results between sexes is because female have more WMH [56]. The sex difference is also particularly pronounced for periventricular WMH [57]. Thus, high blood sugar levels may be an important risk factor for cognitive function in women. In conclusion, the antioxidant effects of high SUA may be particularly important for elderly female participants with high FPG level and oxidative stress and low antioxidant defenses. Men have a higher risk of cerebrovascular diseases than women [58]; as such, the higher SUA-related cerebrovascular burden may counteract the antioxidant effects of high SUA, which may explain why the results were not significant in men.

This study has some advantages. First, the results provided additional evidence to explore the potential relationship of FPG and SUA to cognitive decline. Second, CHARLS contained a number of possible confounders potentially affecting cognitive function, and these covariables were reasonably adjusted through multi-step analysis. Third, in addition to quaternized SUA and FPG, sensitivity analyses were conducted for the combination of DM and low SUA, thereby improving the robustness of the results. However, some limitations should be considered. First, this work was an observational study. Thus, no clear cause-and-effect relationship could be obtained. Second, the included individuals had significantly higher educational levels and cognitive scores than the excluded CHARLS participants (Table S1). Thus, our findings are suggestive only for participants with better cognitive and educational levels. Third, measurement of SUA and FPG levels only once may not be sufficient to accurately estimate a representative concentration level for a person over time. Fourth, although cognitive function in several cognitive domains was measured, it may still be relatively limited and the effect of SUA/blood glucose levels on other cognitive domains, such as executive function, remains unclear. Finally, we lacked information on the structure of the high-purine diet of the participants.

Lower SUA was associated with poorer cognitive function in women with higher blood sugar levels. The small β in our study is different from previous studies, suggesting that a change in SUA of one quartile is associated with a small change in cognitive function, which may not be clinically applicable but can be predictive. Further research is warranted to examine whether or not combined interventions for controlling SUA in a slightly higher range and FPG in a slightly lower range could translate into clinical benefits for the protection of cognition.

## Supplementary Information

**Additional file 1.**

## Data Availability

The data for this study were downloaded from the CHARLS data repository, http://charls.pku.edu.cn/.

## Abbreviations

*FPG*: Fasting plasma glucose
*D**M*: Diabetes mellitus
*S**U**A*: Serum uric acid
*TICS*: Telephone Interview of Cognitive Status
*B**MI*: Body mass index
*W**MH*: White matter hyperintensities

## References

[1] WHO Dementia: a public health priority. http://www.who.int/mental_health/publications/demeatia_report_2012/en/ (accessed 3).

[2] Boyle PA, Wang T, Yu L (2021). To what degree is late life cognitive decline driven by age-relatedneuropathologies?. Brain.

[3] Sachdev PS, Lipnicki DM, Kochan NA (2015). The Prevalence of Mild Cognitive Impairment in Diverse Geographical and Ethnocultural Regions: The COSMIC Collaboration. PLoS One.

[4] Jia L, Du Y, Chu L (2020). Prevalence, risk factors, and management of dementia and mild cognitive impairment in adults aged 60 years or older in China: a cross-sectional study. Lancet Public Health.

[5] Mijailovic NR, Vesic K, Borovcanin MM (2022). The Influence of Serum Uric Acid on the Brain and Cognitive Dysfunction. Front Psychiatry.

[6] Xue L, Liu Y, Xue H (2017). Low uric acid is a risk factor in mild cognitive impairment. Neuropsychiatr Dis Treat.

[7] Scheepers L, Jacobsson LTH, Kern S (2019). Urate and risk of Alzheimer's disease and vascular dementia: a population-based study. Alzheimers Dement.

[8] Bowman GL, Shannon J, Frei B (2010). Uric acid as a CNS antioxidant. J Alzheimers Dis.

[9] Xue M, Xu W, Ou YN (2019). Diabetes mellitus and risks of cognitive impairment and dementia: a systematic review and meta-analysis of 144 prospective studies. Ageing Res Rev.

[10] Lacy ME, Gilsanz P, Eng C (2020). Severe hypoglycemia and cognitive function in older adults with type 1 diabetes: the study of longevity in diabetes (SOLID). Diabetes Care.

[11] Rawlings AM, Sharrett AR, Albert MS (2019). The association of late-life diabetes status and hyperglycemia with incident mild cognitive impairment and dementia: the ARIC study. Diabetes Care.

[12] Pappas C, Small BJ, Andel R (2019). Blood glucose levels may exacerbate executive function deficits in older adults with cognitive impairment. J Alzheimers Dis.

[13] DeBosch BJ, Kluth O, Fujiwara H (2014). Early-onset metabolic syndrome in mice lacking the intestinal uric acid transporter SLC2A9. Nat Commun.

[14] Feig DI, Kang DH, Johnson RJ (2008). Uric acid and cardiovascular risk. N Engl J Med.

[15] Pfister R, Barnes D, Luben R (2011). No evidence for a causal link between uric acid and type 2 diabetes: a Mendelian randomisation approach. Diabetologia.

[16] Cheng F, Yin X, Duan W (2019). Different-shaped curves for serum uric acid with and without diabetes: results from China health and retirement longitudinal study. J Diabetes.

[17] Zhao Y, Hu Y, Smith JP (2014). Cohort profile: the China Health and Retirement Longitudinal Study (CHARLS). Int J Epidemiol.

[18] Association AD, Standards of medical care in diabetes--2010. Diabetes Care 2010, 33 Suppl 1 (Suppl 1), S11–61. doi:10.2337/dc10-S011. 10.2337/dc10-S011 PMC2797382 20042772

[19] Euser SM, Hofman A, Westendorp RG (2009). Serum uric acid and cognitive function and dementia. Brain.

[20] Khan AA, Quinn TJ, Hewitt J (2016). Serum uric acid level and association with cognitive impairment and dementia: systematic review and meta-analysis. Age (Dordr).

[21] Ekblad LL, Rinne JO, Puukka P (2017). Insulin Resistance predicts cognitive decline: an 11-year follow-up of a nationally representative adult population sample. Diabetes Care.

[22] Zheng F, Yan L, Yang Z (2018). HbA(1c), diabetes and cognitive decline: the English longitudinal study of ageing. Diabetologia.

[23] Xiu S, Zheng Z, Liao Q (2019). Different risk factors for cognitive impairment among community-dwelling elderly, with impaired fasting glucose or diabetes. Diabetes Metab Syndr Obes.

[24] Maiuolo J, Oppedisano F, Gratteri S (2016). Regulation of uric acid metabolism and excretion. Int J Cardiol.

[25] Chung HY, Baek BS, Song SH (1997). Xanthine dehydrogenase/xanthine oxidase and oxidative stress. Age (Omaha).

[26] Bortolotti M, Polito L, Battelli MG (2021). Xanthine oxidoreductase: One enzyme for multiple physiological tasks. Redox Biol.

[27] Lima WG, Martins-Santos ME, Chaves VE (2015). Uric acid as a modulator of glucose and lipid metabolism. Biochimie.

[28] Allegrini S, Garcia-Gil M, Pesi R (2022). The Good, the Bad and the New about Uric Acid in Cancer. Cancers (Basel).

[29] Watanabe K, Watanabe T, Otaki Y (2021). Gender Differences in the Impact of Plasma Xanthine Oxidoreductase Activity on Coronary Artery Spasm. J Clin Med.

[30] Li X, Song P, Li J (2015). Relationship between hyperuricemia and dietary risk factors in Chinese adults: a cross-sectional study. Rheumatol Int.

[31] Wang H, Lou Y, Ma Y (2019). Estimating the glomerular filtration rate and tubular dysfunction in an elderly population with normoalbuminuria in China. Clin Chim Acta.

[32] Davies KJ, Sevanian A, Muakkassah-Kelly SF (1986). Uric acid-iron ion complexes. a new aspect of the antioxidant functions of uric acid. Biochem J.

[33] Ames BN, Cathcart R, Schwiers E (1981). Uric acid provides an antioxidant defense in humans against oxidant- and radical-caused aging and cancer: a hypothesis. Proc Natl Acad Sci U S A.

[34] Álvarez-Lario B, Macarrón-Vicente J (2010). Uric acid and evolution. Rheumatology (Oxford).

[35] Wang X, Wang W, Li L (2013). Oxidative stress and mitochondrial dysfunction in Alzheimer's disease. Biochem Biophys Acta.

[36] Zhang N, Shu HY, Huang T (2014). Nrf2 signaling contributes to the neuroprotective effects of urate against 6-OHDA toxicity. PLoS One.

[37] Kim JW, Byun MS, Yi D (2020). Serum uric acid, Alzheimer-related brain changes, and cognitive impairment. Front Aging Neurosci.

[38] Hayden MR, Tyagi SC (2002). Intimal redox stress: accelerated atherosclerosis in metabolic syndrome and type 2 diabetes mellitus. Atheroscleropathy Cardiovasc Diabetol.

[39] Williamson JR, Chang K, Frangos M (1993). Hyperglycemic pseudohypoxia and diabetic complications. Diabetes.

[40] Beckman JS, Koppenol WH (1996). Nitric oxide, superoxide, and peroxynitrite: the good, the bad, and ugly. Am J Physiol.

[41] Yang J (2019). The role of reactive oxygen species in angiogenesis and preventing tissue injury after brain ischemia. Microvasc Res.

[42] Kim KA, Shin YJ, Kim JH (2012). Dysfunction of endothelial progenitor cells under diabetic conditions and its underlying mechanisms. Arch Pharm Res.

[43] Dhalla NS, Shah AK, Tappia PS (2020). Role of Oxidative Stress in Metabolic and Subcellular Abnormalities in Diabetic Cardiomyopathy. Int J Mol Sci.

[44] Hayden MR, Tyagi SC (2004). Uric acid: A new look at an old risk marker for cardiovascular disease, metabolic syndrome, and type 2 diabetes mellitus: the urate redox shuttle. Nutr Metab (Lond).

[45] Sanguinetti SM, Batthyány C, Trostchansky A (2004). Nitric oxide inhibits prooxidant actions of uric acid during copper-mediated LDL oxidation. Arch Biochem Biophys.

[46] Bellanti F, Matteo M, Rollo T (2013). Sex hormones modulate circulating antioxidant enzymes: impact of estrogen therapy. Redox Biol.

[47] Miller AA, Drummond GR, Mast AE (2007). Effect of gender on NADPH-oxidase activity, expression, and function in the cerebral circulation: role of estrogen. Stroke.

[48] Thambisetty M, Beason-Held L, An Y (2010). APOE epsilon4 genotype and longitudinal changes in cerebral blood flow in normal aging. Arch Neurol.

[49] Shi L, Du X, Zhou H (2014). Cumulative effects of the ApoE genotype and gender on the synaptic proteome and oxidative stress in the mouse brain. Int J Neuropsychopharmacol.

[50] Hsu M, Dedhia M, Crusio WE (2019). Sex differences in gene expression patterns associated with the APOE4 allele. F1000Res.

[51] Damoiseaux JS, Seeley WW, Zhou J (2012). Gender modulates the APOE ε4 effect in healthy older adults: convergent evidence from functional brain connectivity and spinal fluid tau levels. J Neurosci.

[52] Han X, Zhang R, Anderson L (2014). Sexual dimorphism in rat aortic endothelial function of streptozotocin-induced diabetes: possible involvement of superoxide and nitric oxide production. Eur J Pharmacol.

[53] Provenzano FA, Muraskin J, Tosto G (2013). White matter hyperintensities and cerebral amyloidosis: necessary and sufficient for clinical expression of Alzheimer disease?. JAMA Neurol.

[54] Makino T, Umegaki H, Suzuki Y (2014). Relationship between small cerebral white matter lesions and cognitive function in patients with Alzheimer's disease and amnestic mild cognitive impairment. Geriatr Gerontol Int.

[55] Segura B, Jurado MA, Freixenet N (2009). Microstructural white matter changes in metabolic syndrome: a diffusion tensor imaging study. Neurology.

[56] Alqarni A, Jiang J, Crawford JD (2021). Sex differences in risk factors for white matter hyperintensities in non-demented older individuals. Neurobiol Aging.

[57] de Leeuw FE, de Groot JC, Achten E (2001). Prevalence of cerebral white matter lesions in elderly people: a population based magnetic resonance imaging study. The Rotterdam Scan Study. J Neurol Neurosurg Psychiatry.

[58] Appelros P, Stegmayr B, Terént A (2009). Sex differences in stroke epidemiology: a systematic review. Stroke.

