# Supplementary material for: Association of serum uric acid and fasting plasma glucose with cognitive function: a cross-sectional study
Source: BMC Geriatr. 2023 May 5;23:271. doi: 10.1186/s12877-023-03998-9 (PMC10161633; doi:10.1186/s12877-023-03998-9)
Supplement: Supplementary file 1 — Additional file 1. [file 12877_2023_3998_MOESM1_ESM.docx]

Supplementary Material

# Supplementary Tables

Table S1: Baseline characteristics.

| Characteristics | Excluded  (n=11196 ) | Included | | | | *P_2_* value |
| --- | --- | --- | --- | --- | --- | --- |
|  |  | All participants (n=6509) | Male (n=3257) | Female (n=3252) | *P_1_* value |  |
| Age (years), mean (SD) | 59.71 ± 10.86 | 57.98 ± 8.70 | 58.87 ± 8.76 | 57.08 ± 8.54 | **<0.001** | **<0.001** |
| Male (n, %) | 5219 (46.61) | 3257 (50.03) | 3257 (100.00) | 0 (0.00) | - | **<0.001** |
| FPG, mmol/L | 6.16 ± 2.15 | 6.10 ± 2.01 | 6.14 ± 2.06 | 6.07 ± 1.96 | 0.136 | 0.147 |
| SUA, mg/dL | 4.39 ± 1.24 | 4.52 ± 1.28 | 5.01 ± 1.31 | 4.04 ± 1.05 | **<0.001** | **<0.001** |
| Educational level |  |  |  |  | **<0.001** | **<0.001** |
| Primary school or lower | 7912 (70.67) | 3895 (14.86) | 1707 (52.41) | 2188 (67.28) |  |  |
| Middle school | 2005 (17.91) | 1647 (25.30) | 967 (29.69) | 680 (20.91) |  |  |
| High school or above | 1279 (11.42) | 967 (59.84) | 583 (17.90) | 384 (11.81) |  |  |
| Current married | 9583 (85.82) | 5917 (90.90) | 3041 (93.37) | 2876 (88.44) | **<0.001** | **<0.001** |
| BMI, kg/m^2^ | 23.26 ± 3.91 | 23.91 ± 3.91 | 23.28 ± 3.73 | 24.54 ± 3.98 | **<0.001** | **<0.001** |
| Smoking status |  |  |  |  | **<0.001** | **<0.001** |
| Current | 2819 (25.18) | 2043 (31.39) | 1849 (56.77) | 194 (5.97) |  |  |
| Former | 803 (7.17) | 614 (9.43) | 557 (17.10) | 57 (1.75) |  |  |
| Never | 7574 (67.65) | 3852 (59.18) | 851 (26.13) | 3001 (92.28) |  |  |
| Alcohol consumption |  |  |  |  | **<0.001** | **<0.001** |
| Current | 3494 (31.21) | 2274 (34.94) | 1880 (57.72) | 394 (12.12) |  |  |
| Former | 473 (4.22) | 330 (5.07) | 262 (8.04) | 68 (2.09) |  |  |
| Never | 7229 (64.57) | 3905 (59.99) | 1115 (34,24) | 2790 (85.79) |  |  |
| Global Cognition, mean (SD) | 10.13 ± 6.84 | 16.73 ± 4.25 | 17.12 ± 3.95 | 16.35 ± 4.50 | **<0.001** | **<0.001** |
| Episodic Memory, mean (SD) | 4.42 ± 4.14 | 8.17 ± 2.97 | 8.16 ± 2.82 | 8.19 ± 3.12 | 0.656 | **<0.001** |
| Mental Status, mean (SD) | 5.30 ± 3.45 | 8.56 ± 2.31 | 8.97 ± 2.15 | 8.16 ± 2.39 | **<0.001** | **<0.001** |
| Hypertension | 2617 (23.37) | 1667 (25.61) | 809 (24.84) | 858 (26.38) | 0.149 | **0.005** |
| DM | 1255 (11.21) | 1081 (16.61) | 550 (16.89) | 531 (16.33) | 0.545 | **<0.001** |
| Kidney disease | 671 (5.99) | 435 (6.68) | 234 (7.18) | 201 (6.18) | 0.100 | 0.118 |
| Stroke | 296 (2.64) | 117 (1.80) | 64 (1.96) | 53 (1.63) | 0.306 | **<0.001** |
| Heart diseases | 1284 (11.47) | 809 (12.43) | 331 (10.16) | 478 (14.70) | **<0.001** | 0.111 |
| Dyslipidemia | 897 (8.01) | 698 (10.72) | 339 (10.41) | 359 (11.04) | 0.369 | **<0.001** |
| Gastrointestinal disease | 2464 (22.01) | 1436 (22.06) | 654 (20.08) | 782 (24.05) | **<0.001** | **0.015** |
| Liver disease | 416 (3.72) | 260 (3.99) | 135 (4.14) | 125 (3.84) | 0.525 | 0.459 |
| Treatment for kidney disease | 348 (3.11) | 244 (3.75) | 130 (3.99) | 114 (3.51) | 0.302 | **0.030** |
| Diabetes treatment | 431 (3.85) | 281 (4.32) | 125 (3.84) | 156 (4.80) | 0.057 | 0.163 |

Abbreviations: FPG, fasting plasma glucose; SUA, uric acid; DM, diabetes mellitus. *P_1_* **<** 0.05, the gender difference in this characteristic was statistically significant in included; *P_2_* **<** 0.05, the difference in this characteristic between the included and excluded populations was statistically significant. *P* values < 0.05 were highlighted in bold.

Table S2: Association between DM and cognitive decline.

| Variable | Global Cognition | |  | Episodic Memory | |  | TICS | |
| --- | --- | --- | --- | --- | --- | --- | --- | --- |
|  | Model 1  β (95% CI) | Model 2  β (95% CI) |  | Model 1  β (95% CI) | Model 2  β (95% CI) |  | Model 1  β (95% CI) | Model 2  β (95% CI) |
| Non-DM | Ref. | Ref. |  | Ref. | Ref. |  | Ref. | Ref. |
| DM | 0.093  (-0.193, 0.380) | 0.088  (-0.204, 0.380) |  | 0.003  (-0.199, 0.206) | 0.009  (-0.197, 0.216) |  | 0.090  (-0.066, 0.247) | 0.079  (-0.081, 0.239) |

Model 1 adjusted for age, sex, BMI.

Model 2 adjusted for age, sex, BMI, hypertension, stroke, dyslipidemia, heart diseases and smoking status.

Abbreviations: DM, diabetes mellitus; BMI, body mass index.

Table S3: Association between combination of DM and SUA quartiles and cognitive function.

| All participants | Global cognition | | |  | Episodic memory | | |  | Mental status | |
| --- | --- | --- | --- | --- | --- | --- | --- | --- | --- | --- |
|  | Model 1  β (95% CI) | Model 2  β (95% CI) | |  | Model 1  β (95% CI) | | Model 2  β (95% CI) |  | Model 1  β (95% CI) | Model 2  β (95% CI) |
| Non | Ref. | | Ref. |  | Ref. | Ref. | |  | Ref. | Ref. |
| Low SUA | **-0.297**  **(-0.573, -0.020)** | | **-0.322**  **(-0.602, -0.042)** |  | **-0.233**  **(-0.438, -0.038)** | **-0.252**  **(-0.450, -0.055)** | |  | -0.064  (-0.215, 0.087) | -0.069  (-0.222, 0.084) |
| DM | 0.285  (-0.248, 0.817) | | 0.231  (-0.307, 0.769) |  | 0.106  (-0.271, 0.482) | 0.074  (-0.306, 0.455) | |  | 0.179  (-0.112, 0.470) | 0.157  (-0.137, 0.451) |
| Both | -0.362  (-0.751, 0.027) | | -0.378  (-0.772, 0.016) |  | **-0.312**  **(-0.587, -0.037)** | **-0.324**  **(-0.603, -0.046)** | |  | -0.050  (-0.263, 0.163) | -0.054  (-0.269, 0.162) |

Model 1: adjusted for age, sex, BMI.

Model 2: adjusted for age, sex, BMI, hypertension, stroke, dyslipidemia, heart diseases, kidney disease, liver disease, gastrointestinal disease and smoking status.

Abbreviations: DM, diabetes mellitus; SUA, serum uric acid; BMI, body mass index. β_s_ 95% CI without 0 were highlighted in bold.

Table S4: Association between combination of DM and SUA and cognitive function (stratified by sex).

| All participants | Global cognition | |  | Episodic memory | |  | Mental status | |
| --- | --- | --- | --- | --- | --- | --- | --- | --- |
|  | Model 1  β (95% CI) | Model 2  β (95% CI) |  | Model 1  β (95% CI) | Model 2  β (95% CI) |  | Model 1  β (95% CI) | Model 2  β (95% CI) |
| **Male** |  |  |  |  |  |  |  |  |
| Non | Ref. | Ref. |  | Ref. | Ref. |  | Ref. | Ref. |
| Low SUA | **-0.466**  **(-0.804, -0.128)** | **-0.465**  **(-0.806, -0.125)** |  | **-0.360**  **(-0.602, -0.119)** | **-0.365**  **(-0.608, -0.121)** |  | -0.106  (-0.294, 0.083) | -0.101  (-0.290, 0.089) |
| DM | -0.034  (-0.724, 0.656) | -0.219  (-0.915, 0.477) |  | -0.205  (-0.698, 0.288) | -0.326  (-0.824, 0.173) |  | 0.171  (-0.214, 0.556) | 0.107  (-0.281 0.495) |
| Both | -0.077  (-0.550, 0.397) | -0.158  (-0.636, 0.320) |  | -0.148  (-0.486, 0.190) | -0.202  (-0.544, 0.141) |  | 0.071  (-0.193, 0.335) | 0.044  (-0.223, 0.310) |
| **Female** |  |  |  |  |  |  |  |  |
| Non | Ref. | Ref. |  | Ref. | Ref. |  | Ref. | Ref. |
| Low SUA | **-0.626**  **(-1.014, -0.237)** | **-0.667**  **(-1.060, -0.275)** |  | **-0.392**  **(-0.663, -0.121)** | **-0.413**  **(-0.687, -0.140)** |  | **-0.234**  **(-0.443, -0.024)** | **-0.254**  **(-0.466, -0.042)** |
| DM | 0.589  (-0.144, 1.322) | 0.446  (-0.290, 1.181) |  | 0.395  (-0.117, 0.908) | 0.319  (-0.194, 0.833) |  | 0.194  (-0.202, 0.589) | 0.126  (-0.271, 0.523) |
| Both | **-0.758**  **(-1.320, -0.196)** | **-0.800**  **(-1.369, -0.232)** |  | **-0.465**  **(-0.858, -0.073)** | **-0.492**  **(-0.889, -0.095)** |  | -0.292  (-0.595, 0.011) | **-0.309**  **(-0.615, -0.002)** |
| *P* for interaction | 0.150 | 0.227 |  | 0.461 | 0.580 |  | 0.093 | 0.135 |

Model 1: adjusted for age, BMI, hypertension.

Model 2: adjusted for age, BMI, hypertension, stroke, dyslipidemia, heart diseases, kidney disease, liver disease, gastrointestinal disease and smoking status.

Abbreviations: DM, diabetes mellitus; SUA, serum uric acid; BMI, body mass index. β_s_ 95% CI without 0 were highlighted in bold.

Table S5: Association between SUA/FPG and cognitive function.

|  | SUA | |  | FPG | |
| --- | --- | --- | --- | --- | --- |
|  | Model 1  β (95% CI) | Model 2  β (95% CI) |  | Model 1  β (95% CI) | Model 2  β (95% CI) |
| Global cognition | **0.149 (0.057, 0.240)** | **0.148 (0.055, 0.241)** |  | 0.003 (−0.049, 0.056) | −0.001 (−0.057, 0.056) |
| Episodic memory | **0.105 (0.040, 0.170)** | **0.103 (0.037, 0.169)** |  | −0.014 (−0.051, 0.023) | −0.017 (−0.057, 0.023) |
| Mental status | 0.044 (−0.006, 0.094) | 0.045 (−0.006, 0.096) |  | 0.018 (−0.011, 0.046) | 0.017 (−0.014, 0.048) |

Model 1: adjusted for age, sex, BMI.

Model 2_SUA_: adjusted for age, sex, BMI, smoking status, hypertension, dyslipidemia, stroke, heart diseases, kidney disease, liver disease, gastrointestinal disease, DM.

Model 2_FPG_: adjusted for age, sex, BMI, smoking status, hypertension, dyslipidemia, stroke, heart diseases, diabetes treatment.

Abbreviations: SUA, serum uric acid; FPG, fasting plasma glucose; BMI, body mass index; DM, diabetes mellitus. β_s_ 95% CI without 0 were highlighted in bold.

Table S6: Association between SUA/FPG and cognitive function (stratified by sex).

|  | SUA | |  | FPG | |
| --- | --- | --- | --- | --- | --- |
|  | Model 1  β (95% CI) | Model 2  β (95% CI) |  | Model 1  β (95% CI) | Model 2  β (95% CI) |
| **Male** |  |  |  |  |  |
| Global cognition | **0.136 (0.026, 0.246)** | **0.130 (0.019, 0.240)** |  | 0.047 (−0.021, 0.116) | 0.045 (−0.028, 0.117) |
| Episodic memory | **0.086 (0.008, 0.165)** | **0.083 (0.004, 0.162)** |  | 0.007 (−0.041, 0.056) | 0.009 (−0.043, 0.061) |
| Mental status | 0.050 (−0.012, 0.111) | 0.047 (−0.015, 0.108) |  | **0.040 (0.002, 0.078)** | 0.036 (−0.005, 0.076) |
| **Female** |  |  |  |  |  |
| Global cognition | **0.203 (0.045, 0.360)** | **0.198 (0.038, 0.357)** |  | −0.029 (−0.110, 0.052) | −0.049 (−0.136, 0.039) |
| Episodic memory | **0.150 (0.039, 0.260)** | **0.148 (0.036, 0.259)** |  | −0.033 (−0.089, 0.024) | −0.047 (−0.108, 0.015) |
| Mental status | 0.053 (−0.032, 0.138) | 0.050 (−0.036, 0.136) |  | 0.004 (−0.040, 0.048) | −0.002 (−0.049, 0.045) |

Model 1: adjusted for age, BMI, hypertension.

Model 2_SUA_: adjusted for age, BMI, smoking status, hypertension, dyslipidemia, stroke, heart diseases, kidney disease, liver disease, gastrointestinal disease, DM.

Model 2_FPG_: adjusted for age, BMI, smoking status, hypertension, dyslipidemia, stroke, heart diseases, diabetes treatment.

Abbreviations: SUA, serum uric acid; FPG, fasting plasma glucose; BMI, body mass index; DM, diabetes mellitus. β_s_ 95% CI without 0 were highlighted in bold.
